# Risk Factors in the Prediction of Leg Numbness after Spinal Endoscopic Surgery: Evaluation and Development of a Nomogram

**DOI:** 10.1155/2022/9502749

**Published:** 2022-11-08

**Authors:** Ming Yi, Wenjun Wang, Shixin Pan, Shengsheng Huang, Xuhua Sun, Liyi Chen, Chong Liu, Xinli Zhan

**Affiliations:** ^1^Spine and Osteopathy Ward, First Affiliated Hospital of GuangXi Medical University, Nanning, Guangxi Province 53000, China; ^2^The First Affiliated Hospital, Department of Pain, Hengyang Medical School, University of South China, Hengyang, Human, 421001, China; ^3^Department of Spinal Surgery, The First Affiliated Hospital, Hengyang Medical School, University of South China, Hengyang, Human, 421001, China; ^4^Department of Spinal Surgery, Wuzhou Red Cross Hospital, Wuzhou, Guangxi Province, China

## Abstract

**Purpose:**

This study aims at constructing a clinical predictive model that predicted the risk factors for leg numbness after spinal endoscopic surgery.

**Methods:**

We collected the clinical data of patients, including general information, imaging parameters, and clinical score, from our hospital's electronic database. Based on the postoperative leg numbness visual analog scale (LN-VAS), the clinical data were divided into the leg numbness group (≥25) and the improvement group (<25). All parameters were included in the least absolute shrinkage and selection operator (LASSO) regression analysis, while the parameters with the area under the curve (AUC) greater than 0.7 were selected to construct nomograms. Furthermore, the accuracy and validity of the model were evaluated using the C-index, decision curve analysis (DCA), calibration curve, and receiver operating characteristic curve (ROC).

**Results:**

A total of 73 patients' clinical data were included in the training set, where 51 patients were assigned to the improvement group and 22 to the leg numbness group. The nomogram was constructed using four selected parameters, including symptom duration, lumbar spinal stenosis (LSS), pelvic incidence (PI), and preoperative low back pain visual analog scale (LBP-VAS). The nomogram predictions were found to range between 0.01 and 0.99. The values of the C-index, AUC, and internally validated C-index were 0.96, 0.96, and 0.94, respectively. Our result showed that the clinical net benefit of the nomogram ranged between 0.01 and 0.99.

**Conclusion:**

Our clinical prediction model demonstrated high predictive ability and clinical validity. Moreover, we found that symptom duration, LSS, PI, and preoperative LBP-VAS were the predictive risk factors for leg numbness after spinal endoscopic surgery.

## 1. Introduction

Recently, endoscopic spinal surgery has been developing rapidly due to its advantages of less trauma, less bleeding, and shorter hospital stay [1]. It has achieved good results in common single-segment L4/L5 or L5/S1 intervertebral disc herniation [2]. Additionally, spinal endoscopic treatment was reported to display good results in multisegment lumbar disc herniation [3]. The surgical approach to spinal endoscopy is usually divided into two types, namely, the intervertebral foraminal and the interlaminar approaches [4, 5]. Regardless of the surgical approach, leg numbness after spinal endoscopic surgery is common [6].

Despite the several advantages of spinal endoscopy, some postoperative complications of leg numbness are still observed in a few patients [7]. Toyoda et al. performed a cluster analysis on patients, and found that despite an improvement after spinal endoscopic surgery, cluster 4 patients retained severe leg numbness, postoperatively [8]. Yan et al. showed that patients with central disc herniation displayed a longer duration of leg numbness after spinal endoscopic surgery than patients with paracentral disc herniation [9]. Ogura et al. showed that 60% of patients retained leg numbness after spinal endoscopic surgery [10]. Although a few studies have reported risk factors for leg numbness after spinal endoscopic surgery, such reports were based on traditional statistical analyses and not the construction of clinical predictive models [9]. Ogura et al. conducted a study based on multivariate logistic regression analysis, which revealed durotomy, diabetes mellitus, and preoperative numbness to be the risk factors for postoperative leg numbness [10].

In this study, we constructed a clinical prediction model to investigate the risk factors for leg numbness after spinal endoscopic surgery. Additionally, we used C-index, calibration curve, ROC curve, and DCA curve to further evaluate the diagnostic ability and clinical validity of the model. Our nomogram can be considered useful as a new clinical prediction tool.

## 2. Materials and Methods

Clinical data for this study were collected between January 2021 and December 2021. The inclusion criteria for lumbar disc herniation were as follows: (1) lower back and lower extremity pain, (2) a positive result in the straight leg raising test, (3) imaging results confirming the nucleus pulposus herniation and compression of the nerve, and (4) patients showing no improvement after half a year of conservative treatment. The exclusion criteria were as follows: (1) lumbar instability, (2) lumbar spine tumors, (3) lumbar tuberculosis, and (4) infectious diseases of the spine. All patients displayed L4/L5 disc herniation and unilateral nerve root compression. To confirm the presence of nerve damage in the lower extremities, nerve conduction studies were performed in all patients before and after surgery. According to the postoperative leg numbness VAS, patients were divided into two groups, namely, the leg numbness (≥ 25) and the improvement group (< 25). Our study used LN-VAS, which is a visual pain analog scale (0-100 mm horizontal analog scale) commonly used for leg numbness. Several reports in the literature have also evaluated the efficacy of LN-VAS after spinal endoscopic surgery [7, 9, 11].

### 2.1. Data Collection

All patients' data were obtained from our hospital's electronic database and imaging system. First, we collected the general information, including gender, body mass index (BMI), hypertension, diabetes, and symptom duration. Next, we gathered the patients' imaging parameters, including lumbar disc grade, degree of LSS, endplate disease grade, degree of endplate disease, lumbar lordosis angle, pelvic incidence, lumbar spondylolisthesis, adjacent lumbar disc grade, and lumbar scoliosis. Finally, we collected the patients' clinical scores, including preoperative and postoperative JOA, preoperative and postoperative LBP-VAS, preoperative and postoperative leg pain VAS (LP-VAS), and preoperative and postoperative LN-VAS. Previously reported literature was used to refer to the criteria for grading clinical data, which included the degree of LSS, degree of endplate disease, LBP-VAS, LP-VAS, and LN-VAS [11–13].

### 2.2. Statistical Analysis

R software (version 4.1) and SPSS software (version 28.0) were used for statistical analysis and plotting of the data. The clinical scores were jointly calculated by two senior surgeons from the spinal department, while the imaging measurements were jointly decided by two senior physicians from the imaging department. A value of *P* less than 0.05 was considered statistically significant.

## 3. Results

**Figure 1:**
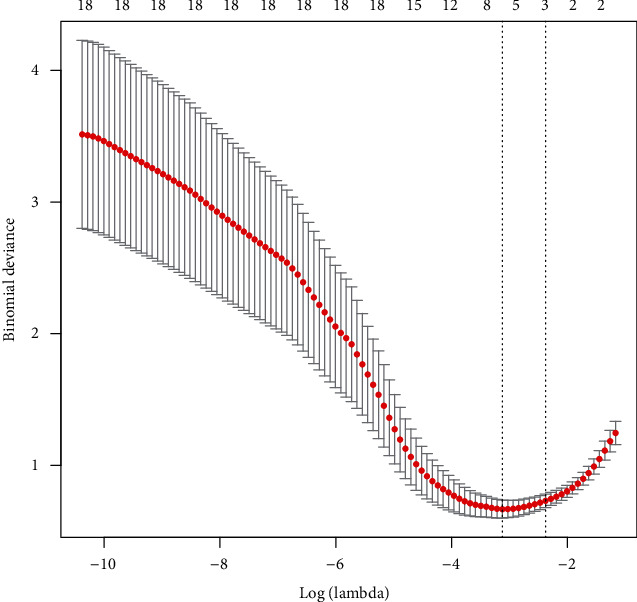
Minimum binomial deviance obtained by LASSO regression analysis.

**Figure 2:**
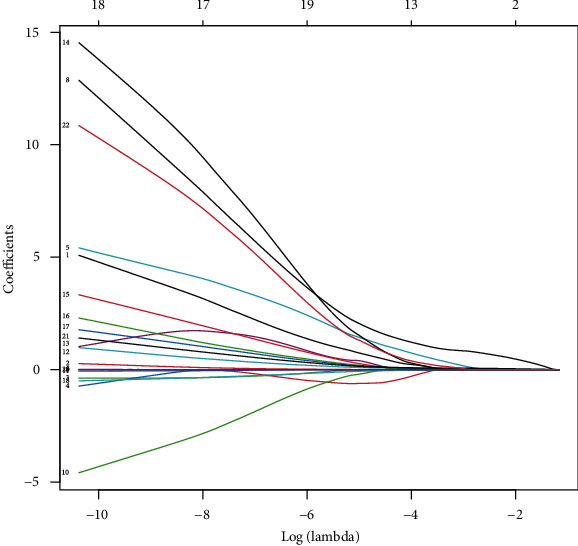
LASSO regression analysis screening the parameters with nonzero coefficients along with the optimal lambda value.

**Figure 3:**
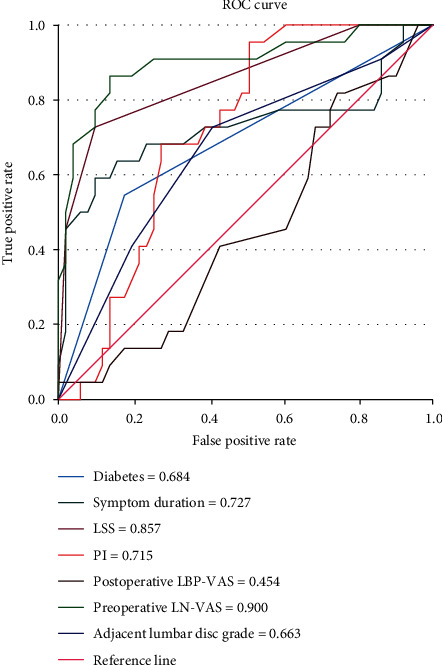
Symptom duration, LSS, PI, and preoperative LBP-VAS belonged to the parameters with AUC greater than 0.7, obtained by ROC curve analysis.

We collected the clinical data from a total of 102 patients undergoing spinal endoscopic treatment in our hospital. In this study, we performed a follow-up period of 6 months to 12 months after endoscopic surgery. In the training set, 22 patients retained leg numbness after surgery (leg numbness group), while 51 patients showed no obvious leg numbness after surgery (improvement group) (Table 1). These patients had leg numbness before surgery. In the external validation set, 10 patients retained leg numbness after surgery, while 19 patients showed no obvious leg numbness after surgery. To obtain the smallest binomial deviance (Figure 1) and the optimal lambda value (Figure 2), all parameters were included in the LASSO regression analysis. Under the optimal lambda value conditions, seven nonzero coefficient parameters were fitted, which were included in the ROC curve analysis. Here, the parameters with AUC greater than 0.7, including symptom duration, LSS, PI, and preoperative LBP-VAS, were selected to construct a nomogram (Figure 3).

**Figure 4:**
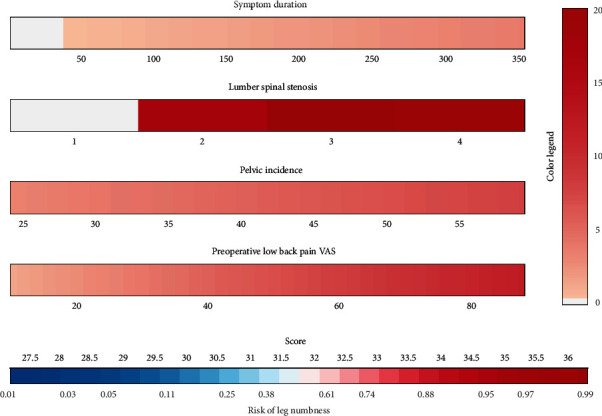
The clinical predictive model predicting leg numbness after spinal endoscopic surgery.

**Figure 5:**
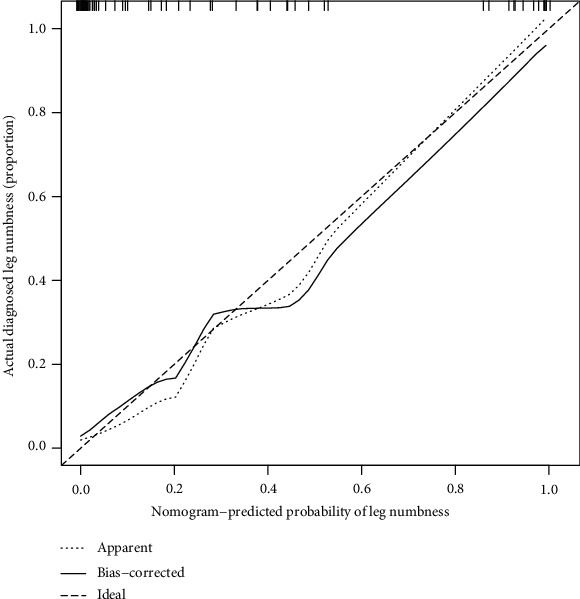
The calibration curve showing almost similar observed and ideal values.

**Figure 6:**
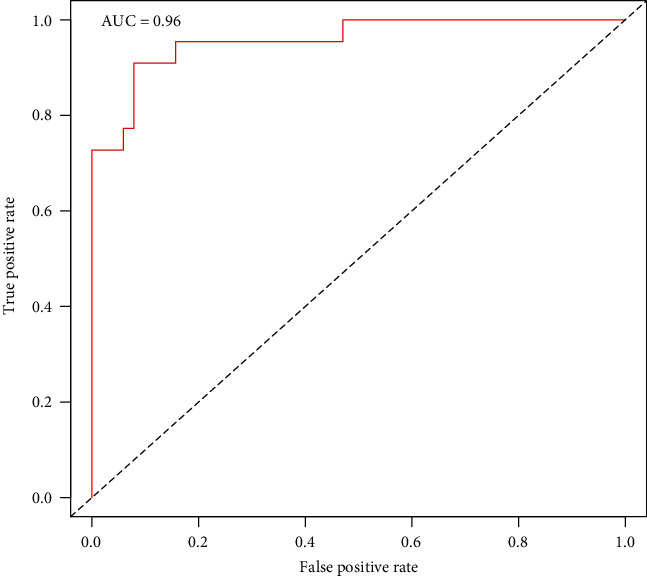
The ROC curve analysis showing the AUC of the clinical prediction model to be as high as 0.96.

**Figure 7:**
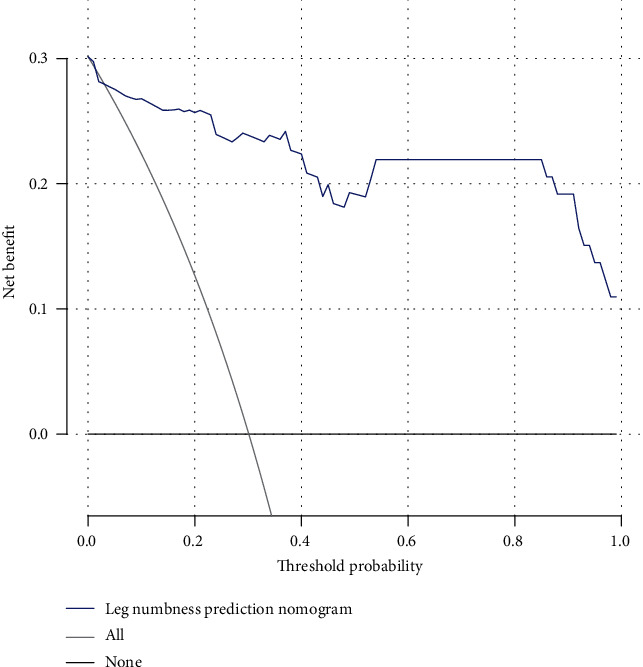
The net benefit of the model in clinical practice ranging between 0.01 and 0.99.

**Figure 8:**
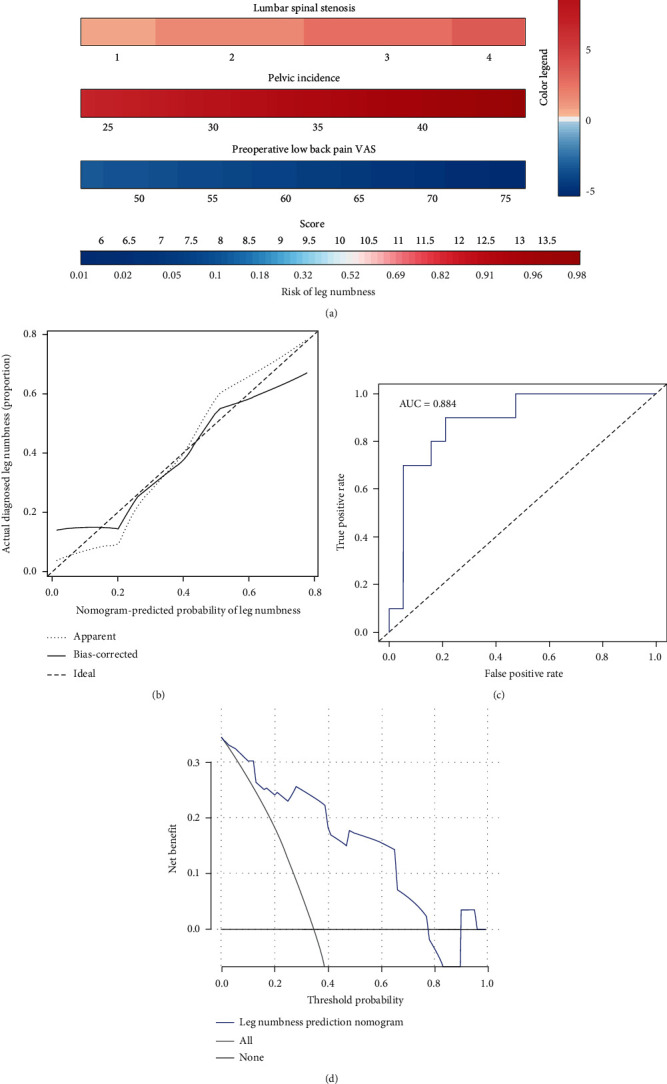
External validation for the evaluation of the predictive model. (a) The predictors in the clinical prediction model included symptom duration, LSS, PI, and preoperative LBP-VAS. (b) Calibration curve. (c) ROC curve. (d) DCA curve.

The nomogram indicated that the prediction of postoperative leg numbness ranged between 0.01 and 0.99 (Figure 4), while the C-index was found to be as high as 0.96. The calibration curve showed a similarity in both observed and ideal values (Figure 5). Additionally, the ROC curve showed the AUC of the nomogram to be as high as 0.96, indicating a strong diagnostic ability of the predictive model (Figure 6). Our results showed that the clinical net benefit of the nomogram ranged between 0.01 and 0.99 (Figure 7). Furthermore, the internal validation results demonstrated that the C-index of the nomogram was as high as 0.94, which was very close to the C-index of 0.96 in the training set. Interestingly, the model in the external validation set also displayed a good predictive ability (Figure 8(a)). Moreover, the ideal value of the calibration curve was close to the actual observed value (Figure 8(b)). The AUC of the ROC curve and C-index both showed a value of 0.884 (Figure 8(c)). Further, internal verification showed that the C-index was as high as 0.747, while the DCA curve indicated the clinical validity of the predictive model to range between 0.01 and 0.77 (Figure 8(d)).

**Figure 9:**
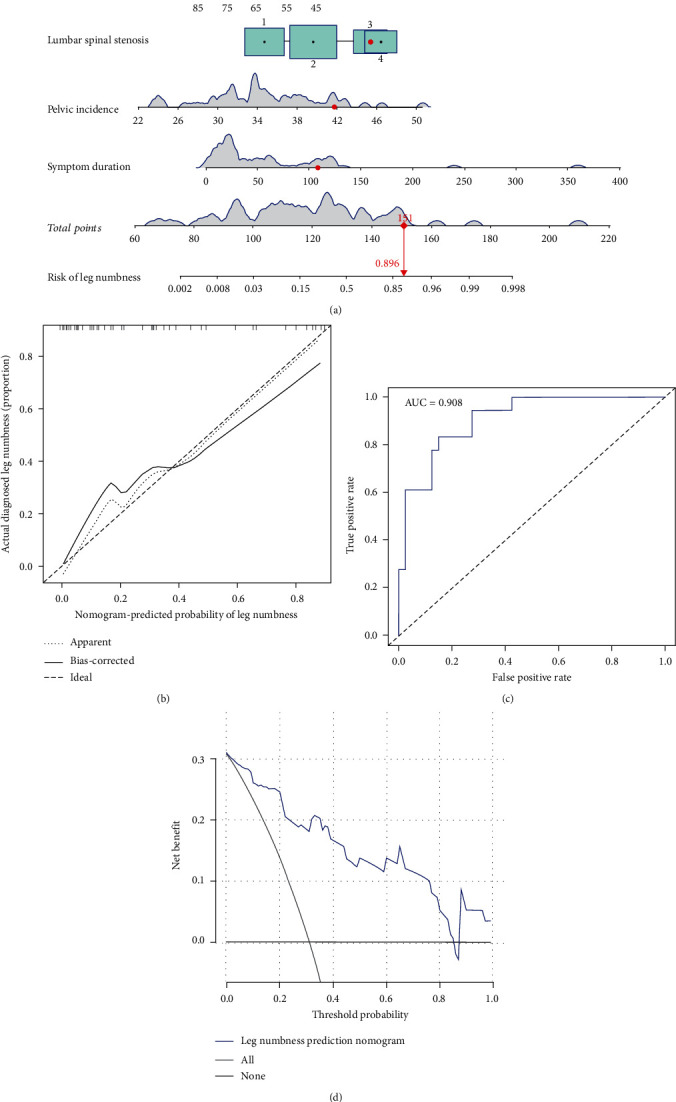
A prediction model of leg numbness constructed based on the transforaminal approach. (a) Clinical prediction model. (b) Calibration curve. (c) ROC curve. (d) DCA curve.

**Figure 10:**
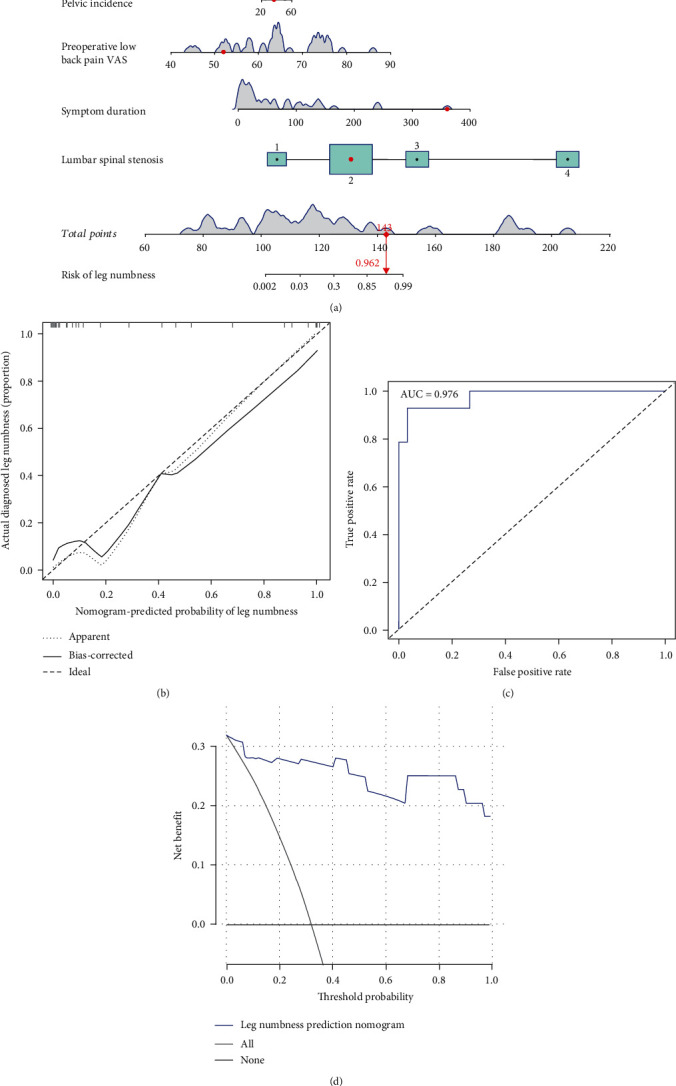
A prediction model of leg numbness constructed based on the interlaminar approach. (a) Clinical prediction model. (b) Calibration curve. (c) ROC curve. (d) DCA curve.

Furthermore, the surgical approach for all data was divided into transforaminal and interlaminar approaches. In the transforaminal approach, symptom duration, LSS, PI, and preoperative LBP-VAS were used as predictors to construct the clinical prediction model (Figure 9(a)). The bias-correction curve was found to be very close to the ideal curve (Figure 9(b)). The AUC and C-index of the prediction model both showed a value of 0.908 (Figure 9(c)). Interestingly, the internal verification of C-index was found to be 0.850, while the DCA curve indicated a clinical efficacy ranging between 0.01 and 0.85 (Figure 9(d)). In the interlaminar approach, the nomogram was constructed using the same four parameters as in transforaminal approach. This nomogram also displayed a good predictive ability (Figure 10(a)). The calibration curve showed that the predicted value of the model was very close to the ideal value (Figure 10(b)). The AUC and C-index of the nomogram both showed a value of 0.976 (Figure 10(c)). Interestingly, the internal verification of the C-index value was 0.942, while the DCA curve indicated a clinical efficacy ranging between 0.01 and 0.99 (Figure 10(d)).

## 4. Discussion

Recently, endoscopic spinal surgery has been vastly favored by young patients with intervertebral disc herniation [14]. Compared to traditional open surgery, endoscopic spinal surgery displays the advantages of less trauma, less bleeding, and shorter hospital stays [1]. Although the validity of spinal endoscopic surgery is remarkable, the complications of postoperative leg numbness cannot be ignored [15]. Most of the previous studies conducted on the validity and risk factors of spinal endoscopic surgery are based on traditional statistical analysis. Hence, we used a novel clinical prediction model to study the risk factors of spinal endoscopic surgery. First, we analyzed all the parameters using LASSO regression. The optimal lambda values obtained by controlling the parameters with nonzero coefficients were used to construct a clinical prediction model. Next, the accuracy and validity of the model were evaluated using the C-index, ROC curve, DCA curve, and calibration curve.

Our results revealed symptom duration to be a predictor of leg numbness after spinal endoscopic surgery. The longer the symptom duration, the higher the probability of predicting leg numbness. In an analysis of 241 patients with postoperative complications after spinal endoscopic surgery, Shen et al. observed retained leg numbness in 176 patients. These patients displayed a common longer symptom duration, ranging from 33.5 ± 52.7 to 36.3 ± 61.9 months [16]. A longer symptom duration in patients indicated that the nerve root was compressed for a long time, and even after the surgery to relieve nerve compression, the recovery time taken by such patients was longer compared to patients with shorter symptom duration. This may be the reason for some patients retaining leg numbness after spinal endoscopy surgery. However, with the extension of follow-up time, the complications of leg numbness were alleviated gradually or even disappeared eventually. In our study, symptom duration exerted an effect on postoperative leg numbness and was considered its predictor.

LSS was also found to be a predictor of leg numbness after spinal endoscopic surgery. The higher the grade of LSS, the higher the probability of predicting leg numbness. Ogura et al. showed that patients with lumbar spinal stenosis who underwent spinal endoscopic surgery were less satisfied and also complained of residual leg numbness after surgery [17]. Similarly, Yamamoto et al. showed that the leg numbness VAS in the unsatisfactory patients after LSS surgery was as high as 64.0 ± 23.6, while the leg numbness VAS in the satisfied patients was 33.7 ± 29.6, with both groups showing a statistically significant difference [18]. The literature has reported seven cases of nerve root damage and two cases of residual numbness in patients with severe spinal stenosis who underwent spinal endoscopy [19]. Interestingly, our study identified LSS as a risk factor for postoperative leg numbness, suggesting that surgeons should properly communicate with patients before performing LSS surgery.

Furthermore, PI was found to be a predictor of leg numbness after spinal endoscopic surgery. The higher the PI, the higher the probability of predicting leg numbness. Only a few studies have reported an association between PI and leg numbness, with a positive correlation between PI and lumbar lordosis [20]. Moreover, when the matching relationship between PI-LL was maintained, patients achieved the best surgical results after spinal surgery [21]. Increased lumbar lordosis can result in a loss of posterior intervertebral space height and reduced foraminal volume, which can be the cause of leg numbness. After surgery, segmental lordosis showed an improvement of 29%, while foraminal height increased from 7.7% to 29.9% [22]. After restoring the height of the intervertebral space, the volume of the intervertebral foramen increased by 39.6%, resolving the leg numbness caused by nerve root compression [23]. In addition, spinal sagittal imbalance caused by degenerative scoliosis also caused leg numbness and PI-LL was found to be significantly positively correlated with SVA (*r* = 0.647, *P* < 0.001) [24]. The study of Aoki et al. also found that PI-LL was significantly correlated with the occurrence of leg numbness after spinal endoscopy, which indicated that PI-LL mismatch would affect the symptoms of leg numbness after surgery [25]. Therefore, before undergoing spinal endoscopic surgery, attention should be paid to patients with reduced foraminal volume caused due to increased PI.

Preoperatively, LBP-VAS is considered a predictor of leg numbness after spinal endoscopic surgery. Taiji et al. showed the leg numbness VAS to be as high as 61.4 to 65.2 in patients with lower back pain after endoscopic spinal surgery [11]. A multiple logistic regression study also showed preoperative lower back pain to be a risk factor (OR = 2.19) for postoperative lower back pain [11]. A herniated disc destroys the annulus fibrosus, releasing chemicals that irritate the sinus nerve and cause lower back pain [26]. In addition, a herniated disc compresses the nerve roots directly, resulting in leg numbness [27]. Therefore, disc herniation often causes lower back pain and leg numbness. Since the nerves of patients with preoperative lower back pain are already compressed, the severity of the nerve compression displays more obvious symptoms of lower back pain. Although spinal endoscopic surgery relieved nerve compression, the nerve roots took a longer time to recover in patients already having severe low back pain. Therefore, some patients still showed leg numbness after spinal endoscopic surgery. Our results also showed that the higher the preoperative LBP-VAS, the higher the probability of predicting leg numbness, which was consistent with the mentioned studies.

However, our study had some limitations since it included single-center data. Studies with multicenter data may be required for further validation.

## 5. Conclusion

We constructed a clinical prediction model to predict leg numbness after endoscopic surgery, which exhibited high accuracy and practicability. Our study revealed that symptom duration, LSS, PI, and preoperative LBP-VAS were the risk factors predicting leg numbness after spinal endoscopic surgery.

## Figures and Tables

**Table 1:** Comparison of clinical information between the leg numbness and the improvement groups.

Characteristics	Improvement (*N* = 51)	Leg numbness (*N* = 22)
Gender		
Female	26 (51%)	10 (45%)
Male	25 (49%)	12 (55%)
Age		
Mean ± SD	59.6 ± 15.7	62.0 ± 11.1
BMI		
Mean ± SD	22.5 ± 2.92	25.0 ± 3.43
Hypertension		
No	35 (69%)	15 (68%)
Yes	16 (31%)	7 (32%)
Diabetes		
No	42 (82%)	10 (45%)
Yes	9 (18%)	12 (55%)
Symptom duration		
Mean ± SD	43.9 ± 44.5	120 ± 105
Lumbar disc grade		
I	2 (4%)	0 (0%)
II	22 (43%)	4 (18%)
III	10 (20%)	4 (18%)
IV	17 (33%)	14 (64%)
Degree of LSS		
I	10 (20%)	0 (0%)
II	36 (71%)	6 (27%)
III	4 (8%)	6 (27%)
IV	1 (2%)	10 (45%)
Endplate disease grade		
I	13 (25%)	6 (27%)
II	30 (59%)	8 (36%)
III	8 (16%)	8 (36%)
Degree of endplate disease		
I	29 (57%)	7 (32%)
II	15 (29%)	10 (45%)
III	7 (14%)	5 (23%)
Lumbar lordosis		
Mean ± SD	36.9 ± 7.40	36.2 ± 7.32
Pelvic incidence		
Mean ± SD	37.1 ± 8.44	41.7 ± 5.49
Lumbar spondylolisthesis		
No	43 (84%)	18 (82%)
Yes	8 (16%)	4 (18%)
Lumbar scoliosis		
No	41 (80%)	12 (55%)
Yes	10 (20%)	10 (45%)
Preoperative JOA		
Mean ± SD	15.1 ± 3.42	14.6 ± 3.97
Postoperative JOA		
Mean ± SD	24.7 ± 2.45	23.7 ± 3.51
Preoperative LBP-VAS		
Mean ± SD	64.1 ± 9.15	68.0 ± 9.17
Postoperative LBP-VAS		
Mean ± SD	22.6 ± 9.32	21.3 ± 9.35
Preoperative LP-VAS		
Mean ± SD	64.2 ± 12.1	66.8 ± 7.46
Postoperative LP-VAS		
Mean ± SD	20.0 ± 8.70	24.3 ± 11.1
Preoperative LN-VAS		
Mean ± SD	35.3 ± 11.8	60.0 ± 13.9
Adjacent lumbar disc grade		
I	7 (14%)	2 (9%)
II	23 (45%)	4 (18%)
III	11 (22%)	7 (32%)
IV	10 (20%)	9 (41%)

## Data Availability

The original contributions presented in the study are included in the article; further inquiries can be directed to the corresponding authors.
